# Characterization of Soft Contact Lens Fitting Using Ultra-Long Scan Depth Optical Coherence Tomography

**DOI:** 10.1155/2017/5763172

**Published:** 2017-06-01

**Authors:** Lele Cui, Ming Li, Meixiao Shen, Fan Lu, Jianhua Wang

**Affiliations:** ^1^School of Ophthalmology and Optometry, Wenzhou Medical University, Wenzhou, Zhejiang, China; ^2^Department of Ophthalmology, Bascom Palmer Eye Institute, University of Miami, Miami, FL, USA; ^3^Electrical and Computer Engineering Department, University of Miami, Miami, FL, USA

## Abstract

**Objectives:**

To evaluate the centration and movement of soft contact lenses and to verify the repeatability of two repeated measurements of the lens centration and movement using ultra-long scan depth optical coherence tomography (UL-OCT).

**Methods:**

A 1-day Acuvue® Define™ lens was tested on both eyes of 10 subjects (5 males and 5 females; mean age, 31.6 years). The centration and blink-induced movement of the contact lens were measured using UL-OCT at 5 min and 30 min after insertion. The measurements were repeated once at each checkpoint.

**Results:**

Good repeatability was found in the lens centration and movement between the two repeated measurements at either checkpoint. The values of the lens movement were 0.457 ± 0.248 mm and 0.402 ± 0.229 mm at 5 min and decreased to 0.197 ± 0.065 mm and 0.211 ± 0.110 mm at 30 min after insertion for the right and left eyes, respectively (*P* < 0.05).

**Conclusions:**

The custom-built UL-OCT presented good repeatability of centration and movement in Define lenses at 5 min and 30 min after insertion. Most of the lenses were centered temporal and inferior to the cornea during the first 30 min wearing period. Compared with 5 min after insertion, the lens was centered better and exhibited less movement at 30 min.

## 1. Introduction

The soft contact lens has gradually become one of the most common approaches employed to correct refractive errors since its initial breakthrough in the early 1960s by Czech chemists Wichterle and Lim [1] and its approval for marketing by the United States FDA in 1971 [2]. However, soft contact lens wear has been associated with certain changes to the ocular surface because of the close contact with the eye, which indicates the importance of the assessment of the lens fitting during clinical practice. Discontinuation of contact lens wear occurs partially as a result of inappropriate lens fitting [3–6], which may have negative impacts on the ocular physiology, such as greater fluorescein staining and high levels of bulbar hyperemia [7].

Descriptions of contact lens in-eye performance have been reported by several researchers [8–10]. Among these descriptions, lens movement and centration are two critical aspects in the adequacy of lens fitting. Lens motion is an indicator of mixing in the postlens tear film [11, 12], which is a useful gauge of the uptake of the epithelial oxygen [13] and the removal of debris and dead cells [14, 15]. Analogously, lens decentration may compromise the optimal visual outcome [16] and is associated with incomplete corneal coverage [17] and corneal staining [18].

The Define lens is a cosmetic contact lens, designed to change the appearance of the eye but also able to correct refractive error. Similar to any other contact lenses, cosmetic lenses carry risks of complications, including ocular redness, irritation, and infection [19, 20]. Because of the potential danger in these colored lenses, which are printed with dye, anyone who would like to wear cosmetic lenses should have a rigorous fitting examination prior to usage.

With the rapid development of optical coherence tomography (OCT), high-speed spectral domain OCT was demonstrated to be capable of quantifying lens movement [21]. This study aimed to characterize the centration and movement of the Define lens and to verify the repeatability of two repeated measurements in the lens centration and movement using ultra-long scan depth OCT (UL-OCT).

## 2. Subjects and Methods

### 2.1. Subjects and Materials

This study was approved by the Institutional Review Boards of the University of Miami and conducted at the Bascom Palmer Eye Institute. The study was conducted in accordance with the tenets of the Declaration of Helsinki. Informed consent was obtained from each participant after a full explanation of the procedures but before enrollment in the study. The patients were screened prior to the study participation to ensure no involvement of previously diagnosed dry eye or dry eye symptoms or any pathology that would normally contraindicate contact lens wear. During screening, slit-lamp biomicroscopy evaluation was performed to confirm the lens fitting with a centration of less than 1 mm [22, 23] and a blink-induced movement within 0.5 to 1.0 mm [24]. After a screening test, 10 subjects (5 males and 5 females; mean ± standard deviation of age, 31.6 ± 7.3 years) were recruited for the study. The 1-day Acuvue Define lenses (etafilcon A, 58% water content, 8.5 mm base curve, 14.2 mm diameter, +0.00 power; Accent™, Vistakon, Johnson & Johnson, Jacksonville, FL) were used during this study.

### 2.2. Optical Coherence Tomography Instrument

A custom-built, spectral domain, ultra-long scan depth OCT (UL-OCT) was used to image the centration and blink-induced movement of contact lenses. The instrument was described in detail in our previous study [21, 25]. In brief, a superluminescent diode light source (Inphenix, IPSDD 0808; Livermore, CA, USA) was used with a center wavelength of 840 nm and a bandwidth of 50 nm. The power of the incident light delivered into the anterior segment was adjusted to less than 1.30 mW, which was within the safe range to the human eye according to ANSI Z136.1 [26]. The axial resolution of the system was approximately 6.0 *μ*m in the eye, and the scan depth was 7.308 mm in air. The image width was set to 18 mm. Two-dimensional cross-sectional scans (B-scans) were used to image the ocular surface with the lens in place, and the number of A-scans in each B-scan was set to 2048 pixels for imaging. X-Y cross aiming was designed and applied to ensure precise alignment during OCT scanning. In addition, an internal fixation target displayed on a miniature liquid-crystal display (LCD) monitor was provided.

### 2.3. Study Procedure

The study comprised 1 visit for each subject. A 1-day Acuvue Define lens was used. Both eyes of each subject were evaluated. One eye was randomly selected to be examined first, and testing of the companion eye followed in a consulting room with controlled temperature (15–25°C) and humidity (30%–50%) [27] after 10 AM to avoid an edematous state of the cornea and the sleep-induced alteration of the tear film [28–30]. The centration and blink-induced movement of the contact lens were measured using ultra-long scan depth OCT at 5 min and 30 min after insertion, and the measurements were repeated once at each checkpoint.

During the OCT imaging, the subjects were asked to fixate on a target that was straight ahead immediately after every blink and during the eye-open period. The “combined X-Y scanning mode” was used for the evaluation of the lens centration. This mode meant that once the eyes wearing lenses were aligned, OCT images at the horizontal and vertical meridians were obtained through a single acquisition. To visualize lens movement induced by blinking, one acquisition that included 128 continuous frames was imaged at the vertical meridian for 2.7 seconds while blinking, which was similar to the method described in our previous study [21].

### 2.4. Image Processing

**Figure 1:**
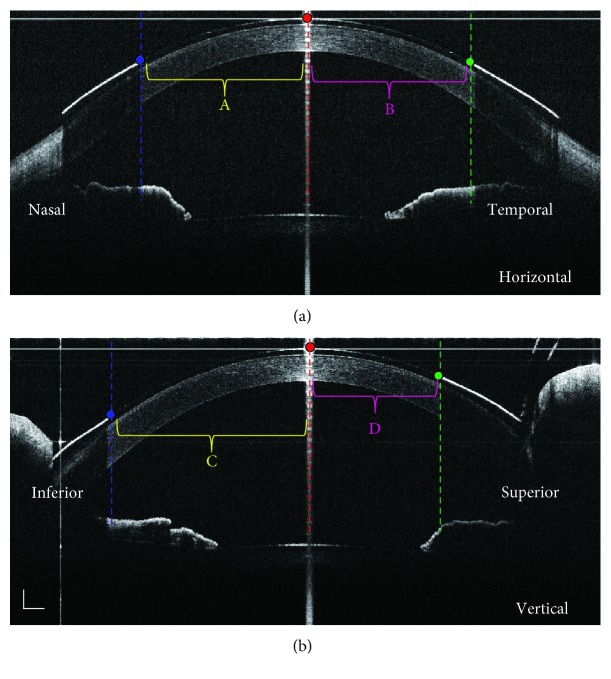
Evaluation of the Define lens centration using UL-OCT images. (a) Horizontal meridian. (b) Vertical meridian. The nasal (blue dot)/temporal (green dot)/inferior (blue dot)/superior (green dot) boundaries of the inner painted pupil/iris in lenses were indicated. The perpendicular distances between those four points and the corneal apex (red dashed lines) were marked as “A,” “B,” “C,” and “D.” The difference between “A” and “B” was defined as the decentration at the horizontal meridian (a). The difference between “C” and “D” was defined as the decentration at the vertical meridian (b). The bars indicate 500 *μ*m.

**Figure 2:**
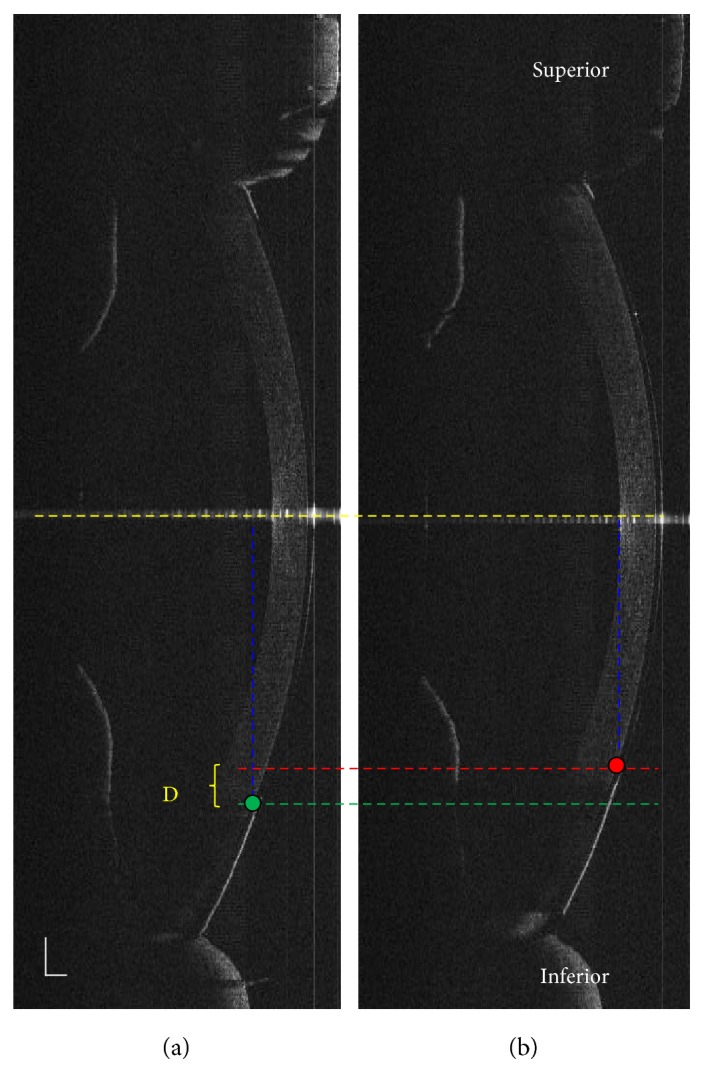
Assessment of the Define lens movement through UL-OCT images. (a) was before a blink and (b) after. The lens movement was tracked as the perpendicular distance between red/green dots and the yellow dashed line. The linear distance “D” was defined as the amount of the lens movement. The bars indicate 500 *μ*m.

All of the variables in lens location and movement were measured by a series of custom software programs. We tracked the lens centration with respect to the corneal apex. First, the nasal/temporal/inferior/superior boundaries of the inner painted pupil/iris in lenses were indicated. Then, the difference of the perpendicular distance between the painted points and the corneal apex at both horizontal and vertical meridians were calculated (Figure 1). For the right eye, positive values indicated that the lens was located nasal to the corneal apex, and negative values indicated that the lens was positioned temporally. The horizontal variables of the lens centration were opposite for the left eyes. With respect to the vertical meridian, positive values indicated that the lens was located inferior to the corneal apex, and negative values indicated that the lens was positioned superiorly. The amount of lens movement was assessed as the perpendicular difference between the inferior inner painted points to the apex before blinking compared with the same distance after blinking (Figure 2).

### 2.5. Data Analysis

Data analysis was conducted using the Statistical Package for Social Sciences (SPSS version 17.0 for Windows XP; SPSS Inc., Chicago, IL, USA). All of the data were presented as the means ± standard deviations. The mean values and the average differences between the repeated measurements were calculated. Repeated measures analysis of variance (Re-ANOVA) was performed to determine the differences between repeated measurements. The intraclass correlation coefficient (ICC), which was determined on the basis of the analysis of variance for two-way mixed effects model, was used to assess the reliability of the repeated measurements. The ICC ranges from 0 to 1, and a larger ICC indicates a smaller variability between repeated measurements within a subject [31]. In addition, Bland-Altman plots were used to assess the agreement of the measurements. Paired *t*-tests were applied to analyze the differences of dimensions in the lens centration and movement between the 5 min and 30 min after insertion. *P* < 0.05 was considered to be significant.

## 3. Results

**Figure 3:**
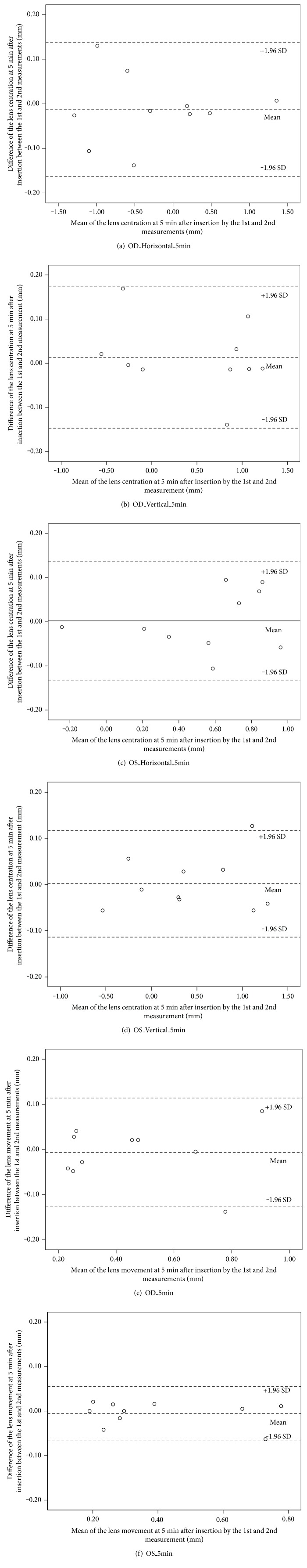
Bland-Altman plots of the differences between two measurements of the lens centration and movement at 5 min after insertion. The mean differences of the variables between two repeated measurements for lens centration (a)–(d) at both meridians and the blink induced (e)-(f). The mean differences in the values between the two measurements were around approximately zero, and the 95% confidence limits were represented by the dotted lines. OD: the right eye; OS: the left eye.

**Figure 4:**
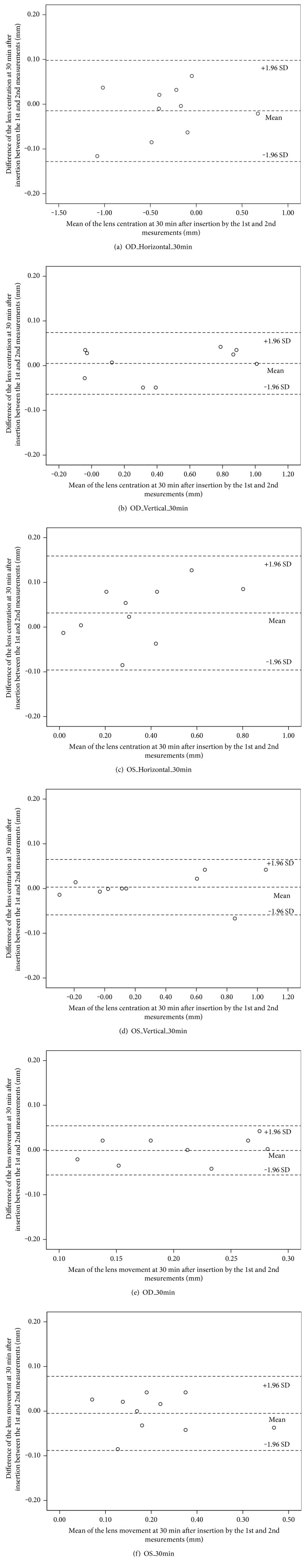
Bland-Altman plots of the differences between two measurements of the lens centration and movement at 30 min after insertion. The mean differences of the variables between two repeated measurements for lens centration (a)–(d) at both meridians and the blink induced (e)-(f). The mean differences in the values between the two measurements were around zero. OD: the right eye; OS: the left eye.

There were no parameters that differed significantly between the two repeated measurements of the lens centration and movement at both checkpoints (Re-ANOVA, *P* > 0.05, Tables 1 and 2). The ICC values were larger than 0.9 for all of the variables, which indicated good repeatability. The Bland-Altman plots showed that the mean differences in values between the two repeated measurements were evenly dispersed around zero, and most of them were within the range of the 95% limits of agreement, which were defined as the mean difference ± 1.96 SD of the differences (Figures 3 and 4).

**Figure 5:**
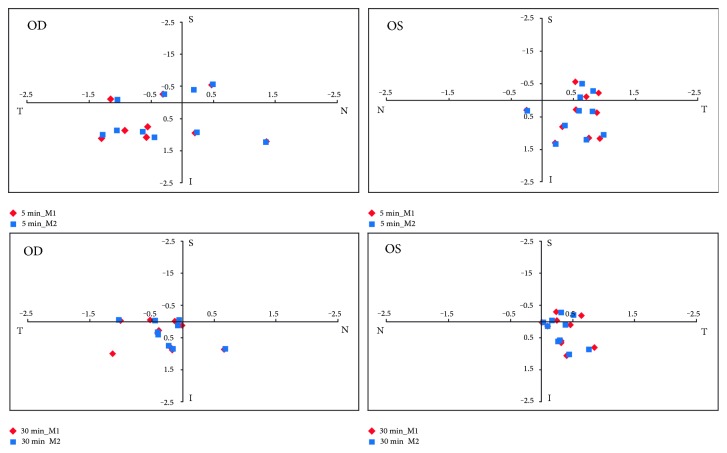
Scatter plots of the lens centration at 5 and 30 min. The lenses were centered temporal and inferior to the corneal apex during the first 30 minutes. Better centration and less movement occurred at 30 min. For the right eye, positive values were denoted with “N” (nasal) and “I” (inferior), and negative with “T” (temporal) and “S” (superior). The horizontal variables of the lens centration were opposite for the left eyes. OD: the right eye; OS: the left eye.

The lenses were positioned temporal and inferior to the corneal apex throughout the first half-hour wearing period (Figure 5). The detailed data regarding the lens centration are listed in Table 3. The lens movements were 0.457 ± 0.248 mm and 0.402 ± 0.229 mm at 5 min after insertion for the right and left eyes, respectively. The amounts of the lens movement were decreased to 0.197 ± 0.065 mm and 0.211 ± 0.110 mm, respectively, at 30 min after insertion. The evaluation of the fitting revealed that better centration and less movement occurred at 30 min after insertion, compared with the behavior of the lens at 5 min after insertion (paired *t*-tests, *P* < 0.05, Table 3 and Figure 5).

## 4. Discussion

Lens centration and movement are two typical measurements in the initial fitting procedure and the aftercare examinations. These measurements are closely associated with ocular health and lens comfort [13–15, 18]. In the present study, we aimed to describe the fitting characteristics of the Define lens and test the repeatability of the measurements using UL-OCT. In the fitting assessment, the centration and movement of lenses were typically measured using a slit lamp with a reticulated eyepiece [8, 32, 34] or captured by video images [16]. In these methods, the limbus and the inferior lens edge were used as references for the evaluation of the lens decentration and the movements, respectively [8, 10, 32–34]. However, the limbus is the anatomical transition zone between the cornea and sclera, which is not a clear-cut boundary and cannot be precisely judged [8]. Additionally, the superior and inferior lens edges and limbus were always covered by the upper and lower eyelids; [8] thus, subjects' lids had to be pulled either upward or downward away from the lens in the fitting procedure [32–34]. This manipulation may cause changes in the lens position (typically allowing it to slip downward), which, in turn, might affect the results. In the present study, lenses with a painted pupil/iris were imaged by UL-OCT to assess centration and movement characteristics, during which movement of the lids was avoided. We tracked the lens centration and movement with respect to the corneal apex, which was clearly observed in the OCT images. This method may minimize the bias. Although ordinary soft lenses do not feature a painted pupil, a fiducial mark might be introduced to the lens design and fitting assessment [35]. In addition, blinking induced lens movement can be tracked using OCT by tracking the lower lens edge [21].

For the repeatability in the measurement of soft lens centration and movement, there is no currently published result using UL-OCT available in the literature. Good repeatability and agreement of the lens centration and movement measurements at 5 min and 30 min after insertion were found based on ICCs and Bland-Altman analysis. It has been suggested that an ICC greater than 0.75 represents good measurement reliability [31]. The lens movement was a dynamic procedure compared with the lens centration; thus, it may exhibit worse repeatability and agreement between two measurements.

The results of our study had revealed that most of the lenses were positioned temporal and inferior to the corneal apex throughout the 30 min wearing period, which confirmed certain clinical findings [8]. Wake et al. [8] observed that Asian subjects presented more decentration compared with Caucasian subjects. The tighter eyelid against the front surface of the eye because of the epicanthal fold in Asian eyes was considered to be the reason for that outcome. Therefore, the tension of the eyelid may play an important role in the centration of contact lenses. In addition, the back optic zone radii, lens diameter, and corneoscleral topography might affect the lens centration. A sharper corneoscleral junction angle at the nasal quadrant found in recent studies [36] might be associated with the temporal decentering of the lens in the present findings.

The observation of variability in the assessment of the lens fitting suggested that lenses exhibited better centration and less movement during the first 30 min. Our finding was in agreement with previous works [37]. As reported by several studies, the lens requires time to equilibrate or settle on the eye [10, 32]. During this period, the decrease in the amount of lens movement might be explained by certain theories. One possible explanation was the lens dehydration that occurred during lens wearing, with the resultant steepening of the lens base curve and the decline in movement [38–40]. Another explanation was lens adherence induced by the outward flow of the postlens tear film, which was caused by the osmotic difference between the tears underneath and outside the lens [41, 42] or by the blinking procedure [43, 44]. A certain level of lens movement contributes to tear exchange, which can remove debris and dead cells and maintain the physical health of the cornea [13–15]. Therefore, accurate quantification of the lens movement could promote a better understanding of the interaction of the tear film and movement.

There were some limitations in the present study. First, we only measured 10 subjects (20 eyes) using UL-OCT for the lens centration and movement; thus, the sample size was small in this study. However, as a pilot study, this work could lay the foundation for further studies. Our method used in the present study could be suitable to study lens physical adaptation which can be studied in future studies. Second, the boundaries of the inner painted pupil/iris of the lenses were obtained by a semiautomated method, which may lead to error in the image analysis. Further development of the automatic segmentation algorithm may mitigate this problem. Third, contact lens fitting is impacted by several factors; therefore, the results of the fitting evaluation will need to be linked to the characterization of the ocular surface shape (especially the corneoscleral topography) and the postlens tear film in further studies. Fourth, it was noted that the standard deviation of the lens movement and centration was high. The high standard deviation might be the characteristic feature of the lens movement and centration due to the interaction between the lens and ocular surface. This phenomenon of high standard deviation in lens movement induced by blinking was evident in our previous study [21]. Lastly, the goal of this pilot study was to demonstrate the feasibility of a novel method which uses UL-OCT to assess the centration and movement characteristics of contact lenses. The method depends on the landmark highlighted in the OCT images. The Define lenses are cosmetic contact lenses with the painted pupil/iris which meet the requirement for testing. Only one type of lenses was chosen and other lenses with the landmarks could be tested in future studies.

In summary, the custom-built UL-OCT presented good repeatability of Define lens centration and movement at 5 min and 30 min after insertion. Most of the lenses were positioned temporal and inferior to the corneal apex throughout the 30 min wearing period. Compared with 5 min after insertion, the lenses were centered better and exhibited less movement at 30 min.

## Figures and Tables

**Table 1:** The repeatability of the lens centration between two repeated measurements.

Variables	Horizontal meridian				Vertical meridian			
	Mean difference	SD of the differences	ICC	*P*	Mean difference	SD of the differences	ICC	*P*
OD_C_5 (mm)	−0.012	0.077	0.996	0.622	0.013	0.081	0.993	0.621
OD_C_30 (mm)	−0.015	0.058	0.994	0.440	0.005	0.035	0.997	0.625
OS_C _5 (mm)	0.002	0.068	0.984	0.921	0.002	0.059	0.996	0.927
OS_C_30 (mm)	0.032	0.065	0.955	0.158	0.003	0.031	0.996	0.760

OD: the right eye; OS: the left eye; C_5: lens centration at 5 min after insertion; C_30: lens centration at 30 min after insertion; SD: standard deviation; ICC: intraclass correlation coefficients.

**Table 2:** The repeatability of the lens movement between two repeated measurements.

Variables	Mean difference	SD of the differences	ICC	*P*
OD_M_5 (mm)	−0.007	0.061	0.971	0.744
OD_M_30 (mm)	−0.001	0.028	0.903	0.901
OS_M _5 (mm)	−0.005	0.028	0.994	0.555
OS_M_30 (mm)	−0.005	0.042	0.925	0.730

OD: the right eye; OS: the left eye; M_5: lens movement at 5 min after insertion; M_30: lens movement at 30 min after insertion; SD: standard deviation; ICC: intraclass correlation coefficients.

**Table 3:** The comparison of the lens centration and movement at 5 and 30 min after insertion.

Variables	Mean ± SD (mm)		*P*
	5 min	30 min	
OD_C_H	0.703 ± 0.444	0.459 ± 0.364	0.046^∗^
OD_C_V	0.723 ± 0.390	0.449 ± 0.399	0.029^∗^
OS_C_H	0.600 ± 0.263	0.341 ± 0.230	0.014^∗^
OS_C_V	0.623 ± 0.438	0.396 ± 0.369	0.003^∗^
OD_M	0.457 ± 0.248	0.197 ± 0.065	0.006^∗^
OS_M	0.402 ± 0.229	0.211 ± 0.110	0.008^∗^

H: horizontal meridian; V: vertical meridian. Only the magnitudes and not the direction of the lens decentration were taken into consideration in this table. ∗ indicates significance (paired *t*-tests, *P* < 0.05).
